# Prevalence of common mental disorder and associated factors among mothers of under five year children at Arbaminch Town, South Ethiopia, 2019

**DOI:** 10.1371/journal.pone.0257973

**Published:** 2021-09-30

**Authors:** Batala Barsisa, Habtamu Derajew, Kibrom Haile, Gebremeskel Mesafint, Shegaye Shumet

**Affiliations:** 1 Department of Psychiatry, Arba Minch University, Arba Minch, Ethiopia; 2 Amanuel Mental Specialized Hospital, Addis Ababa, Ethiopia; 3 Department of Nursing, College of Health Science, Mizan-Tapi University, Tepi, Ethiopia; 4 Department of Psychiatry, College of Medicine and Health Sciences, University of Gondar, Gondar, Ethiopia; Flinders University, AUSTRALIA

## Abstract

**Background:**

Common mental disorders are the major public healthproblem that affects mothers with young children. Although there were a number of studies done on maternal mental health problems, they were largely focused on perinatal period. However, there is scarcity of information on the magnitude and correlates of these mental health problems beyond perinatal period and due concern is not given mainly in LMICs including our country.

**Objective:**

To assess the prevalence and factors associated with common mental disorderamong mothers of under-five year children at Arbaminch town, South Ethiopia, 2019.

**Methods:**

A community based cross-sectional study was conducted in May and June 2019 at Arbaminch town. A systematic random sampling technique was used to select 776 participants. The Self-Reporting Questionnaire (SRQ-20) was used to assess common mental disorder (CMD). Data was coded and entered in EPIDATA3.1 and analyzed using SPSS version 25. Bivariable and multivariable logistic regression were used to identify factors associated to common mental disorder. P-values less than 0.05 were considered statistically significant and strength of the association was presented by adjusted odds ratio with 95% confidence interval.

**Result:**

The prevalence of common mental disorder among mothers with children aged below five years was 36.6% with (95% CI, 33.2, 39.9). Being single/divorced/widowed [AOR = 3.64, 95% CI:(1.47, 8.99), chronic medical illness [AOR = 3.25, 95% CI:(2.10, 5.04)], exposure to two/more stressful events [AOR = 1.62, 95% CI:(1.11, 2.36)], poor social support [AOR = 2.59, 95% CI:(1.62, 4.14)], mothers living with cigarette smoker husband [AOR = 2.03, 95% CI:(1.19, 3.47)], and mothers physically abused by their spouse [AOR = 2.36, 95% CI:(1.49, 3.74)] were factors associated with common mental disorder.

**Conclusion and recommendation:**

The prevalence of common mental disorder was high among mothers with children aged below five years compared to the general population. Being single/divorced/widowed, chronic medicalillness, exposure to two/more stressful events, poor social support, mothers living with cigarette smoker husbandand physically abuse by their spouse were factors associated with common mental disorder. Early detection and management of these maternal mental health problems is vital for mothers’ wellbeing as well as growth and development of children.

## Introduction

Common mental disorders, including depression, anxiety and medically unexplained physical symptoms can cause a considerable lose in health and functioning [1]. Currently, common mental disorder is one of many public health issues, and globally, 14% of the total disease burden accounted for common mental disorders. Unipolar depressive disorder is the third leading cause of disease burden and predicted to be the leading cause of disease burden worldwideby the year 2030 [2].

On average, one in five adults (17.6%) experienced a common mental disorder within the past 12 months and 29.2% in their lifetime. Females were more likely to exhibit mood or anxiety symptoms [3]. Common mental disorders are undetected throughout Africa as result of multiple system and financial challenges such as insufficient number of mental health professionals, low priority/lack of clear mental health policy, poor health infrastructure and lack of evidence-based and culturally appropriate assessment and interventions [4].

Mental disorders, including common mental disorder, are the leading non-communicable disorder in terms of burden in Ethiopia [5]. Evidences showed that the prevalence of common mental disorders in Ethiopia ranges from 14.9%- 27.6% in variety of population with higher rates among women [6–8]. Studies from different countries showed that the magnitude of common mental disorder among mothers with under five year children was higher than the general population. It was 30.36% in Germany [9], 31% in Scotland [10], 56.2% and 43.8% in rural and urban areas, respectively, in Brazil [11]. Similarly, the prevalence was 20% in Kenya [12], and 28.8% in Tanzania [13] among these mothers.

Mental health problems like depression and anxiety among mothers with young children in LMICs are common but under identified and reported health issues, and leading to a significant impairment in maternal functioning [14, 15]. In addition to mothers mental health problem, their children could have increased risk of behavioral and emotional problems, cognitive delays and psychiatric morbidity later in their life [10, 15–17]. Low in educational level, unintended pregnancy, younger in age, being unmarried, lacking intimate partner empathy and support, intimate partner violence, poor social support, and having history of mental health problems were risk factors for maternal CMDs [15, 18]. It is essential to increase the coverage of evidence-based and low-cost interventions for maternal mental health problems to achieve Millennium Development Goals (MDGs); to improve maternal health, reduce child mortality, promote gender equality and empower women, achieve universal primary education and eradicate extreme poverty and hunger [18].

Even though common mental disorders are highly prevalent among mothers of young children, a little attention is paid to these health problems mainly in low and middle income countries [14, 19]. As a result, it has a negative influence on mothers quality of life, and tends to impair the mothers’ ability to respond to the demands of their young children, which in turn has an adverse consequence on the growth and development of their children [10, 15, 17].

Though mental health problems are one of the leading non-communicable health problems in terms of magnitude and burden in Ethiopia, few studies are conducted on maternal mental health problems. Therefore, this study was focusing in identification of factors which contribute to common mental disorder among mothers with under five children because this group of population is vulnerable to these mental health problems. So, the aim of this study was to assess the prevalence and factors associated with common mental disorder among mothers of under-five year children. The result will help indirectly to prevent the burden of mental health problem for children because of mental health problems of their mother.

## Methods

### Study design and period

A community based cross-sectional study was conducted among mothers of under five year children at Arbaminch town between May and June, 2019.

### Study area

The study was carried out at Arbaminch town, Gamo Zone, South Nation Nationalities and people’s regional (SNNPR) state of Ethiopia. Gamo Zone has two lakes (lake Chamo and Abaya), more than 40 springs, National Nech-sarpark, crocodile market. The Zone is known for its several fruits (banana, apple, mango, papaya and avocado) and fish production.

Arbaminch town (the Capital of Gamo Zone) is located 505 km south of Addis Ababa (the Capital of Ethiopia). According to the Federal Democratic Republic of Ethiopia central statistical Agency [20] the total population of the town was 101,819 (with48,300malesand53,519 females), and the town has 11 kebeles/wards/. Moreover, there was one general governmental hospital, and two health centers, which were serving for health service need of the town population.

### Sample size determination and sampling procedure

The sample size was determined by using single population proportion formula by considering the assumptions of 41.8% prevalence, a study conducted in Ethiopia [21], and 1.96 Z (standard normal distribution), 5% margin of error, 95% CI, 10% non-response rate, and using design effect of 2, the calculated sample size was 785.

The multi-stage cluster sampling technique was used to select study participants. At the beginning from the 11 Kebeles of the town, four kebeles were selected randomly using lottery method. Then all mothers with under -five years of age children living in the selected kebeles at least for six months were interviewed until the estimated sample size became full. Participants who were acutely ill and hearing problems were excluded.

### Measurement

Common mental disorder was measured by self-reporting questionnaire (SRQ-20). This questionnaire has 20 “yes/no” response questions, and the items could assess anxiety, depressive and somatic symptoms in the last 30 days. The SRQ-20 has been tested in numerous settings with varied cut-off points. For example, in the community surveys or primary care with a cut-off point 6 with a specificity of 83.7% and sensitivity of 84.8% was used [22]. The SRQ-20 was validated in Ethiopia at a general population, and a cut-off point six was found to have a sensitivity of 90.7% and specificity of 80.7% [23]. Study showed in Ethiopia reliability of the SRQ-20 at perinatal women was checked (Cronbach’s alpha = 0.88 [24], and the reliability in this study was kappa value of 0.87. The Amharic version of the questionnaire was used in this study and this version was also used by other studies in Ethiopia [21, 24, 25].

The Oslo social support scale (OSS-3) was used to assess level of social support. The sum score ranges from 3–14 with 3 categories: 3–8 = poor social support;9–11 = moderate social support; and strong social support 12–14 [26].

List of threatening event questionnaire (LTE-Q) was used to assess exposure to stressful events. It consists of 12 items with “yes/no” response. The instrument was used to measure major stressful events in the preceding 06 months [27], and have convergent validity in various studies in Ethiopia [7, 28, 29].

#### Substance use history

To examine substance use history, respondents were asked: “Have you ever used any substance in the last three months or in your lifetime?” and the responses were Yes/No [30]. Domestic violence questionnaire was used to assess violence related factors [31].

### Data collection procedures

Data were collected by face-to- face interviews by 5BSc nurses by a means of the Amharic version of the tool for a month.

### Data quality control

The questionnaire was designed in English and translated to Amharic and back to English to maintain consistency. The questionnaire was pretested on 5% of the sample size before one week in the actual data collection time. Training was given for data collectors and supervisors on how to interview and explain unclear questions,and maintaining privacy and confidentiality of the participants.

### Data processing and analysis

Data were entered into Epi-data after checking completeness and then exported to SPSS version 20 for analysis. Descriptive statistics such as frequency, percentage and mean were computed. Bi-variable and multivariable logistic regression analyses were performed to identify associated factors of common mental disorder. Factors associated with common mental disorder were selected during the bivariate analysis with a p-value <0.2 for further analysis in the multivariable logistic regression analysis. In the multivariable logistic regression analysis, the strength of association was evaluated using the adjusted odds ratio with a 95% CI, and a P-value less than 0.05.

## Results

### Socio-demographic characteristics of the participants

A total of 776 participants took part in the study with a response rate of 98.8%. The mean (SD) age of the respondents was 30 (5.9) years, and 262 (33.8%) were in the age range of 25–29 years; 727 (93.7%) were married; 381 (49.1%) were Orthodox Christian; 278 (35.8%) had high school educational level and 430(55.4%) were Gamo by ethnicity. Regarding occupation, 222 (28.6%) of the respondents were government employed. Among those mothers having husbands, 14 (1.9%) mothers’ spouses had no formal education **(Table 1)**.

**Table 1:** Socio-demographic characteristics of mothers of under-five year children at Arbaminch town, South Ethiopia, 2019 (n = 776).

Variable	Frequency	Percent (%)
Age		
<20	15	1.9
20–24	116	14.9
25–29	262	33.8
30–34	206	26.5
> = 35	177	22.8
Marital status		
Married	727	93.7
Separated	22	2.8
Single/divorcee/widowed	27	3.5
Religion		
Orthodox	381	49.1
Protestant	316	40.7
Muslim	54	7.0
Others*	25	3.2
Education status		
No formal education	49	6.3
Primary school	172	22.2
High school	278	35.8
College and above	277	35.7
Ethnicity		
Gamo	430	55.4
Gofa	111	14.3
Wolayta	78	10.1
Oromo	46	5.9
Amhara	49	6.3
Others**	62	8.0
Occupational status		
Merchant	113	14.6
Government employed	222	28.6
Private employed	149	19.2
Student	65	8.4
Daily laborer	47	6.1
housewife	180	23.2
Husband Education status		
No formal education	14	1.9
Primary school	96	12.8
High school	295	39.4
College and above	344	45.9
Husband occupational status		
Merchant	174	23.2
Government employed	254	33.9
Private employed	244	32.6
Daily laborer	54	7.2
Others***	23	3.1

Others

* = Apostle and Jehova witness

** = Konso, Gurage, Tigre and Dawro

*** = student and unemployed.

### Clinical factors among the participants

Of the respondents, 112 (14.4%) had family history of mental illness, and about 123 (15.9%) had chronic medical illness. A small number, 61 (7.9%) of the respondents had mental illness history in the past **(Table 2)**.

**Table 2:** Clinical factors among mothers of under-five year children at Arbaminch town, South Ethiopia, 2019 (n = 776).

Variable	Frequency	Percent (%)
Family history of mental illness		
yes	112	14.4
no	664	85.6
Past history of mental illness		
yes	61	7.9
no	715	92.1
Chronic physical illness		
yes	123	15.9
no	653	84.1

### Psychosocial factors

**Fig 1:**
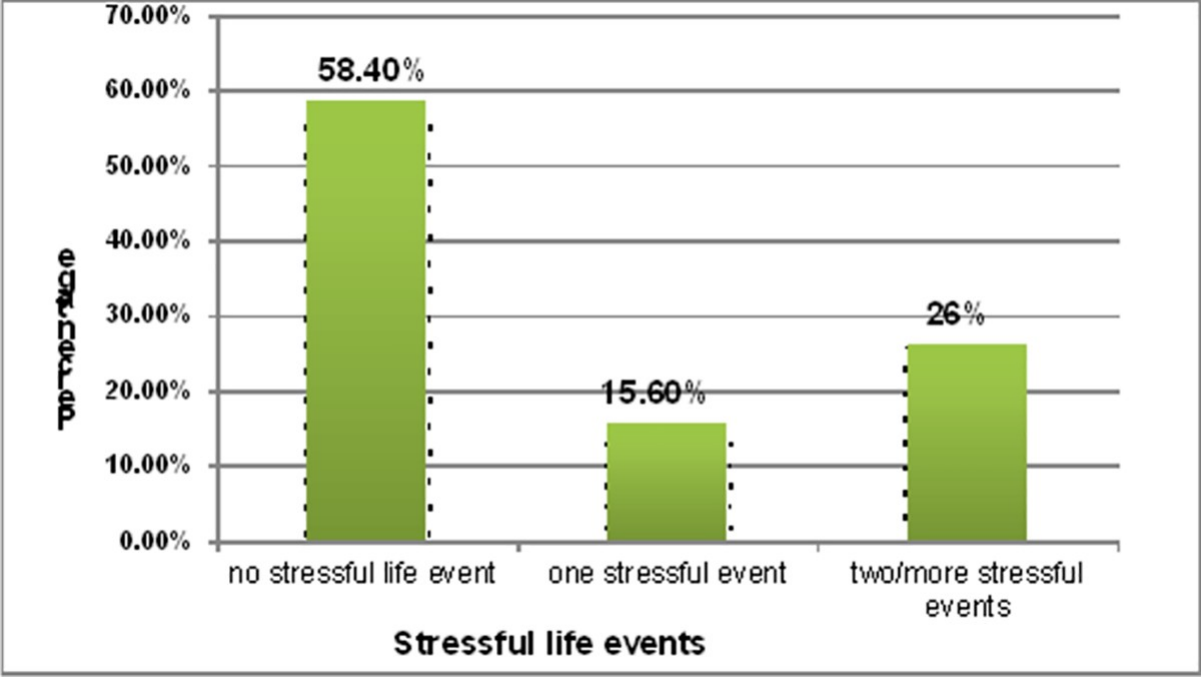
Percentage of stressful life events among mothers of under five year children at Arbaminch town, South Ethiopia, 2019 (n = 776).

Regarding exposure to stressful life events, about 121 (15.6%) of participants had one stressful event and 202 (26%) had two or more stressful events in the preceding 6 months (Fig 1).

Poor social support was reported by 204 (26.3%) of the study participants and 53.20% of them had moderate social support. Of mothers living with their husbands, about 99 (13.2%) reported that their husbands did not assist for caring their child/children. Regarding substance related factors, 239 (30.8%) of mothers consumed alcohol at the moment, 54 (6.96%) were chewing khat (leaves) in the past three months. About 285 (38.1%) husbands were consuming alcohol, 75 (10%) were smoking cigarette, and 146 (19.5%) were using khat (leaves). Nearly half, 374 (49.9%) of mothers faced verbal abuse, and 148 (19.8%) physical abuse by their spouses. About 183 (23.6%) of study participants were pregnant at the moment; 105 (13.5%) of mothers had history of abortion/stillbirth; 64 (8.2%) of mothers reported that their children were dead after birth. 313 (40.3%) of mothers had more than one children in the family **(Table 3)**.

**Table 3:** Substance use characteristics, reproductive factors and marital of mothers of under-five year children at Arbaminch town, South Ethiopia, 2019 (n = 776).

Variable		Frequency	Percent
**Spouse assists with caring for child/children**	yes	650	86.8
	no	99	13.2
**Spouse drinks alcohol**	yes	285	38.1
	no	464	61.9
**Spouse smokes cigarettes**	yes	75	10.0
	no	674	90.0
**Spouse chews chat**	yes	146	19.5
	no	603	80.5
**Experienced verbal abuse by spouse**	yes	374	49.9
	no	375	50.1
**Experienced physical abuse by spouse**	yes	148	19.8
	no	601	80.2
**Perceived marital relationship**	Good	598	79.8
	poor	151	20.2
**Mothers ever use of alcohol**	yes	313	40.3
	no	463	59.7
**Mothers ever use of khat**	yes	83	10.7
	no	693	89.3
**Ever cigarette use**	yes	14	1.8
	no	762	98.2
**Current alcohol use**	yes	239	30.8
	no	537	69.2
**Current khat use**	yes	54	7.0
	no	722	93.0
**Current cigarette use**	yes	4	0.5
	no	772	99.5
**Pregnancy**	yes	183	23.6
	no	593	76.4
**Number of under five year children**	One	463	59.7
	More than one	313	40.3
**Age of the smallest child (in months)**	<13	181	23.3
	13–24	203	26.2
	25–36	197	25.4
	> = 37	195	25.1
**Pregnancy of child**	wanted	575	74.1
	unwanted	201	25.9
**History of abortion/stillbirth**	no	671	86.5
	yes	105	13.5
**History of child death**	no	712	91.8
	yes	64	8.2

### Prevalence of common mental disorder among the respondents

The prevalence of common mental disorder among mothers with under five year children was 36.6% with 95% CI (33.2, 39.9). From the items of the tool, frequent headache (47.2%) and feeling easily tired (49.9%) were the most common symptoms reported by mothers.

### Factors associated with common mental disorder

In the bivariate analysis, socio-demographic variables (marital status and educational level), clinical factors (history of mental illness in the past and chronic physical illness), psychosocial factors (exposure to stressful life events, social support and mothers spent their time in caring of their children alone, husband’s cigarette smoking, physically abused mothers by their spouse and perceived marital relationship), current alcohol use and reproductive/obstetric factors (number of under five children and unwanted pregnancy) were factors associated with common mental disorder at p<0.2 and entered into multivariable logistic regression model for further analysis.

The result of multivariable analysis showed that single/divorced/widowed, mothers with chronic physical illness, exposure to two or more stressful life events, poor social support, spouse who smoked cigarettes and physically abused mothers by their spouse were found to be statistically significant at p-value less than 0.05. The odds of developing common mental disorder among single/divorcee/widowed mothers were 3.64 times higher compared to those who were married [AOR = 3.64, 95% CI: (1.47, 8.99)].

Mothers who had chronic physical illness were 3.25 times more likely to develop common mental disorder compared to their counterparts [AOR = 3.25, 95% CI: (2.10, 5.04)]. The respondents who were exposed to two or more stressful life events in the preceding 06 months were 1.62 times more likely to develop common mental disorder compared to non exposed to stressful events [AOR = 1.62, 95% CI: (1.11, 2.36)]. The odds of developing common mental disorder among mothers with poor social support was 2.59 times higher compared to those with strong social support [AOR = 2.59, 95% CI: (1.62, 4.14)]. Mothers living with their spouses who smoked cigarette were high likely to develop common mental disorder [AOR = 2.03, 95% CI: (1.19, 3.47)]. Mothers who were physically abused by their spouse were 2.36 times more likely to develop common mental disorder [AOR = 2.36, 95% CI: (1.49, 3.74)] **(Table 4)**.

**Table 4:** Bivariable and multivariable logistic regression analysis of associated factors of common mental disorder among mothers of under five year children at Arbaminch town, South Ethiopia, 2019 (n = 776).

Variables	CMD		COR (95% CI)	AOR (95% CI)
	Yes	No		
Marital status				
Married	254	473	1	1
Separated	11	11	1.86(0.80, 4.36)	1.72(0.68–4.35)
Single/divorced/widowed	19	8	**4.42(1.91, 10.25)**	**3.64(1.47, 8.99)** *
Education status				
No formal education	22	27	1.75(0.94, 3.24)	1.85(0.95, 3.59)
Primary school	71	101	1.51(1.02, 2.24)	1.28(0.83, 1.98)
High school	103	175	1.26(0.89, 1.80)	1.26(0.86, 1.85)
College and above	88	189	1	1
Past history of mental illness				
yes	29	32	1.64(0.97, 2.76)	1.68(0.95, 2.97)
no	255	460	1	1
Chronic physical illness				
yes	82	41	**4.47(2.96, 6.73)**	**3.25(2.10, 5.04)** **
no	202	451	1	1
stressful life events in past 06months				
No stressful event	140	313	1	1
One stressful event	39	82	1.06(0.69, 1.64)	0.90(0.57, 1.44)
Two/more stressful events	105	97	**2.42(1.72, 3.40)**	**1.62(1.11, 2.36)** *
Social Support				
Poor	112	92	**2.99(1.93, 4.64)**	**2.59(1.62, 4.14)** **
Moderate	126	287	1.08(0.72, 1.61)	1.07(0.70, 1.63)
Strong	46	113	1	1
Spouse assists with caring for child/children				
yes	212	438	1	1
no	53	46	2.38(1.55, 3.65)	1.58(0.98, 2.53)
Spouse smokes cigarettes				
yes	46	29	**3.30(2.02, 5.39)**	**2.03(1.19, 3.47)** *
no	219	455	1	1
Experienced physical abuse by spouse				
yes	86	62	**3.27(2.26, 4.74)**	**2.36(1.49, 3.74)** **
no	179	422	1	1
Perceived marital relationship				
Good	185	413	1	1
Poor	80	71	2.52(1.75, 3.62)	1.22(0.76, 1.95)
Current alcohol use				
yes	104	135	1.53(1.12, 2.09)	1.38(0.98, 1.94)
no	180	357	1	1
No. of under five year children				
One	159	304	1	1
More than one	125	188	1.27(0.95, 1.71)	1.30(0.94, 1.80)
Pregnancy of child				
wanted	200	375	1	1
unwanted	84	117	1.35(0.97, 1.87)	1.29(0.90, 1.85)

Note

** = p<0.001

* = p<0.05, Hosmer-Lemeshow test = 0.723.

## Discussion

This result revealed that a significant proportion of mothers were experiencing common mental disorder. The magnitude of common mental disorder in this study was 36.6% with (95% CI: 33.2, 39–9). This finding was lower than 41.8% found in Ethiopia [21], 46.2% in Bangladesh [32], and43.8% in Brazil [11]. The possible reason for the discrepancy might be socio-cultural differences. For example: mandated rest, assistance in tasks from relatives and neighbors, and social recognition of mothers through rituals, gifts or other means are common in Ethiopian culture. Another reason for the variation might be sampling technique used. Convenient sampling technique was used in previous Ethiopian and Bangladesh studies, which might have higher estimates of the prevalence of common mental disorder compared to probability sampling technique used in this study. Moreover; different sample size usage could be the other potential variation. Forexample,1068,264, 288 study participants were involvedin Ethiopian, Bangladesh, and Brazil studies, respectively.

However, the finding of this study was higher than other studies such as 28.8% in Tanzania [13], 20% in Kenya [12], 31% in Scotland [10] and 22.2% in Germany [9]. The variation might be due to difference in the study design used, prospective cohort study was used in Scotland study. The other variation might be due to usages of different instruments across studies. For example, depressive or anxiety symptoms were assessed by using 4-item screening tool patient health questionnaire (PHQ-4) in Germany, which assesses only symptoms experienced in the previous two weeks. Common mental disorder was assessed using selected items from depression, anxiety and stress scale (DASS) in Scotland, and locally validated shona symptom questionnaire (SSQ-14) was used in Tanzania study. Participants residence where samples were drawn could be also another variation for the prevalence of common mental disorder, both urban and rural residents were included in the study carried out in Tanzania. Moreover; use of different cut-off point of the instruments in various studies could be the other potential variation for the result,≥ 8 a cut-off point score for SRQ-20 was used in Kenyan study and so that it might have lower common mental disorder estimation. Sample size difference might also be another reason to the discrepancy, Tanzania (n = 1922), Kenya (n = 429), Scotland (n = 3844) and Germany (n = 6679).

Regarding associated factors single/divorcee/widowed, women with chronic physical illness, exposure to two or more stressful life events, poor social support, husband’s cigarette smoking and physically abused by spouses had statistically significant association with common mental disorder. Single/divorcee/widowed mothers were 3.64 times more likely to have common mental disorder than those who were married. This might be related with single mothers were handling every situation alone and might not have the support they need from a partner. Divorcee/widowed women might lack any assistance from their spouse and every duty fall to them which results in decrease in social contact and become stress full. This is supported by the studies conducted in Kenya [12], Brazil [11] and Germany [9].

The odds of developing common mental disorder among respondents with chronic physical illness were more than three times higher compared to their counterparts. This result is supported by study results in Ethiopia [21]. People with chronic physical illness might preoccupied with worries about the illness i.e the disease would not cured and take medication long time,fear about medication side effects, which imposes the women to stress and self-stigmawhich might contribute to common mental disorder.

With respect to exposure to stressful life events, common mental disorder for mothers who were exposed to two or more stressful events in the preceding six months was 1.62 times higher than for those who didn’t. This is in line with other study findings [33]. Severe and prolonged stress might cause over activation and dysregulation of the hypothalamic pituitary adrenal axis thus inflicting detrimental changes in the brain structure and function. These changes would result in mental health problems like depression, anxiety and substance use problems.

In the current study, respondents with poor social support were 2.59 times more likely to develop common mental disorder than those with strong social support. Women with poor social support might have feelings of neglected, not wanted, which leads participants to socially isolated and emotional disturbances. This result is supported by conducted in Scotland [10] and Germany [9].

Another factor which was significantly associated with mothers’ common mental disorder was husband’s cigarette smoking. Women living with cigarette smoker spouses were high likely to develop common mental disorder than non smokers. This might be related with women’s perceived social stigma about their spouse’s substance use behaviors and family and social responsibilities might fall on mothers. There might also be ongoing concerns regarding physical and psychological wellbeing of the partner. This is similar with study carried out in Tanzania [13].

Mothers who experienced physical abuse by their spouses had increased likelihood for developing common mental disorder. Physically abused women might face emotional problems, neglect, isolation, fear, and an inability to trust, which can lead to lifelong consequences, including low self esteem, depression, and relation difficulties [31]. This finding is in agreement with studies conducted in Tanzania [13] and Germany [9].

### Limitation

Social desirability and recall bias might be the limitations. Since the data collection method was a face-to-face interview which might lead mothers to respond in socially acceptable ways during the process, especially in cases of substance-related questions, history of abortion/stillbirth, physical abuse by their spouse, and marital relationship.

### Conclusion and recommendation

This result showed that the magnitude of common mental disorder among mothers was higher compared to the general population. Factors such as single/divorcee/widowed, chronic medical illness, exposure to two or more stressful life events, poor social support, husband’s cigarette smoking and physical abuse by spouse had statistically significant association with common mental disorder. Mothers with under five children need special attention regarding common mental health problems, particularly mothers with chronic physical illness, and abused by their spouses. It is good to give more emphasis to mothers whose spouse smoked cigarette, low social support, and exposed to stress full life events to improve mothers’ quality of life.

## Ethical consideration

Ethical assurance was obtained from the joint Ethical review committee of University of Gondar and Saint Amanuel Mental Specialized Hospital and submitted to Arbaminch town administration. Written informed consent was obtained from each study participants after explaining the purpose and benefit of the study. The participants were informed that they have the right to refuse participation in the study at any time and refusing to participate will not affect them. The interview with study participants was conducted with strict privacy and confidentiality.

## List of abbreviations

AMSH: Amanuel Mental Specialized Hospital
AOR: Adjusted Odds Ratio
CI: Confidence Interval
CMD: Common Mental Disorder
EPDS: Edinburgh Postnatal Depression Scale
HEWs: Health Extension Workers
HMIS: Health Management Information System
LMIC: Low and Middle Income Countries
LTEQ: List of Threatening Events Questionnaire
MCH: Maternal and Child Health
OR: Odds Ratio
OSS: Oslo Social Support Scale
PHQ: Patient Health Questionnaire
PPD: Postpartum Depression
SD: Standard Deviation
SNNPR: South Nation Nationalities and People’s Region
SPSS: Statistical Package Software for Social Sciences
SRQ: Self Reporting Questionnaire
UOG: University of Gondar
WHO: World Health Organization
